# National Studies as a Component of the World Health Organization Initiative to Estimate the Global and Regional Burden of Foodborne Disease

**DOI:** 10.1371/journal.pone.0140319

**Published:** 2015-12-03

**Authors:** Robin J. Lake, Brecht Devleesschauwer, George Nasinyama, Arie H. Havelaar, Tanja Kuchenmüller, Juanita A. Haagsma, Helen H. Jensen, Nasreen Jessani, Charline Maertens de Noordhout, Frederick J. Angulo, John E. Ehiri, Lindita Molla, Friday Agaba, Suchunya Aungkulanon, Yuko Kumagai, Niko Speybroeck

**Affiliations:** 1 Institute of Environmental Science and Research, Christchurch, New Zealand; 2 Department of Virology, Parasitology and Immunology, Faculty of Veterinary Medicine, Ghent University, Merelbeke, Belgium; 3 Department of Biomedical Sciences, Institute of Tropical Medicine, Antwerp, Belgium; 4 Institute of Health and Society, Faculty of Public Health, Université catholique de Louvain, 1200 Brussels, Belgium; 5 Emerging Pathogens Institute, University of Florida, Gainesville, United States of America; 6 Makerere University, Kampala, Uganda; 7 Division of Information, Evidence, Research and Innovation, WHO Regional Office for Europe, Copenhagen, Denmark; 8 Department of Public Health, Erasmus University Medical Center, Rotterdam, Netherlands; 9 Iowa State University, Ames, United States of America; 10 Johns Hopkins Bloomberg School of Public Health, Baltimore, United States of America; 11 Division of Global Health Protection, Center for Global Health, Centers for Disease Control and Prevention, Atlanta, United States of America; 12 Mel and Enid Zuckerman College of Public Health, University of Arizona, Tucson, United States of America; 13 Institute of Public Health, Health & Environment Department, Food Safety & Nutrition Section, Tirana, Albania; 14 National Drug Authority, Ministry of Health, Kampala, Uganda; 15 International Health Policy Program, Ministry of Public Health, Bangkok, Thailand; 16 Department of Veterinary Medical Science, Graduate School of Agriculture and Life Sciences, The University of Tokyo, Tokyo, Japan; Universidad Nacional de la Plata, ARGENTINA

## Abstract

**Background:**

The World Health Organization (WHO) initiative to estimate the global burden of foodborne diseases established the Foodborne Diseases Burden Epidemiology Reference Group (FERG) in 2007. In addition to global and regional estimates, the initiative sought to promote actions at a national level. This involved capacity building through national foodborne disease burden studies, and encouragement of the use of burden information in setting evidence-informed policies. To address these objectives a FERG Country Studies Task Force was established and has developed a suite of tools and resources to facilitate national burden of foodborne disease studies. This paper describes the process and lessons learned during the conduct of pilot country studies under the WHO FERG initiative.

**Findings:**

Pilot country studies were initiated in Albania, Japan and Thailand in 2011 and in Uganda in 2012. A brief description of each study is provided. The major scientific issue is a lack of data, particularly in relation to disease etiology, and attribution of disease burden to foodborne transmission. Situation analysis, knowledge translation, and risk communication to achieve evidence-informed policies require specialist expertise and resources.

**Conclusions:**

The FERG global and regional burden estimates will greatly enhance the ability of individual countries to fill data gaps and generate national estimates to support efforts to reduce the burden of foodborne disease.

## Introduction

The World Health Organization (WHO) initiative to estimate the global and regional burden of foodborne diseases has four stated objectives, two of which involve actions at a national level [1]:

- To strengthen the capacity of countries in conducting burden of foodborne disease assessments and to increase the number of countries that have undertaken a burden of foodborne disease study.
- To encourage countries to use burden of foodborne disease estimates for cost-effective analyses of prevention, intervention and control measures.

As part of the initiative, the Foodborne Disease Burden Epidemiology Reference Group (FERG) was established in 2007 to provide advice and guidance to WHO [2]. FERG established several task forces to address and deliver parts of the initiative, one of which was the Country Studies Task Force (CSTF). The CSTF commenced work in 2009. To specifically address the second objective above a subgroup, the Knowledge Translation and Policy Group (KTPG), was established in 2010.

This paper describes the process and lessons learned during the conduct of pilot country studies under the WHO FERG initiative. Detailed results from studies by individual pilot countries will be published elsewhere.

## Tools and Resources

The initial activity by the CSTF was to develop of a series of tools and resources to facilitate national burden of foodborne disease studies. These were intended to promote a methodology that would be consistent with the global and regional burden estimates being developed by FERG, in particular estimating burden using the disability-adjusted life-year (DALY) metric, and strengthen capacity to develop science-based policies. These tools and resources were developed by members of the CSTF, as well as commissioned scientists, and will be made available on a WHO website dedicated to the burden of foodborne disease initiative. The tools and resources included:

- Reviews of existing burden of disease studies and protocols [3,4];
- A manual on how to conduct a national burden of foodborne disease study (adapted from the WHO manual on national burden of disease estimation [5]);
- A hazard selection tool, including a listing of priority hazards being addressed by the WHO initiative at the global and regional levels, and guidance for identification of hazards that may be locally important;
- Guidance on data collection, describing the information needed to estimate foodborne burden of disease, and potential sources of data, such as surveillance systems, demographic databases, etc. This tool also suggests contextual information that helps to assess data quality; and,
- FERG Situation Analysis/Knowledge Translation/Risk Communication Manual (SA/KT/RC Manual). A situation analysis report or resource is designed to collect and summarize the contextual information concerning food safety in the country undertaking the national foodborne burden of disease study including policies and practices, capacities, key agencies and actors in the food safety system, and document factors that will affect the development of policies and their implementation. The development of this resource benefitted from previous burden of food- and waterborne disease studies in the Caribbean, under the auspices of the Pan American Health Organization (PAHO) [6]. A WHO Global Foodborne Infections Network capacity building workshop in July 2012 resulted in the creation of 13 issue briefs, with context-specific target audiences, and immediately implementable recommendations. The template for these issue briefs was included in the guidance manual (Dr Enrique Perez, PAHO, pers. comm.).

## Pilot Studies

In 2010 WHO invited countries to express interest in conducting national burden of foodborne disease studies as a pilot process. Countries which expressed interest were sent an overview of a national burden of foodborne disease study from the FERG perspective, and a request for information relevant to the conduct of the study. Following an assessment process undertaken by the Department of Food Safety and Zoonoses (FOS) at WHO headquarters, four countries were selected for pilot studies: Albania, Japan, Thailand, and Uganda. A commencement meeting for the Albanian, Japanese and Thai studies was held in November 2011 and the Ugandan study in March 2012. The studies were supported by ongoing communication between the countries and the CSTF.

## Process

Each pilot country was asked to assemble a team to conduct the study. The members of this team included representatives from government and academic institutions. Early in the process the KTPG recommended that the study team conduct a situation analysis according to the guidelines in the SA/KT/RC Manual, to describe the regulatory and economic status of food safety in the country, identify actors, policies and practices, and generally provide context for the scientific data. This analysis would also identify stakeholders who should be aware of the study, could contribute data and information, and might ultimately use the results of the study to guide decision-making.

The initial step in each study was to identify hazards in the food supply that were relevant to the pilot country. Lists of hazards and associated diseases that were considered of global and regional importance by FERG were provided; each country was able to add hazards considered important from their perspective. Available information was then collated on the incidence of diseases associated with the hazards, as well as data on the prevalence of the hazards in the food supply. These data were summarised, and then attribution of disease burden to foodborne transmission was considered, as data allowed.

The KTPG sought to promote knowledge translation and risk communication throughout the development and implementation of the study. Tools for these processes, as described in the SA/KT/RC Manual, are intended to involve stakeholders from the outset so as to promote ownership, share results with stakeholders, and promote efforts to use the information for developing evidence-based policies.

Here we provide a brief overview of key data and food safety systems associated with each pilot study. As enteric disease is a common outcome of exposure to microbial foodborne hazards, there is a focus on data related to enteric disease.

### Albania

Human health surveillance of foodborne diseases in Albania is led by the Public Health Institute within the Ministry of Health, which collates data supplied by regional departments of public health. An early warning surveillance system operates across all of Albania (similar to the system that operates in Serbia and Macedonia [7]), and the case definitions are the same as for syndromic surveillance under the International Health Regulations. Key indicators of foodborne disease are the annual rates of reported gastrointestinal illness (approximately 56,000 cases per year, approximately 2,000 cases per 100,000 population) and cases reported as food poisoning (approximately 2,800 cases per year, approximately 100 cases per 100,000 population). Food poisoning cases are reported on the basis of assessment by physicians from primary health care as well as hospitalized cases. Etiology for cases in these general disease categories is rarely investigated. Surveillance for parasitic or viral infections is not routine, apart from infection with *Entamoeba histolytica*. Cross sectional studies of faecal samples for viral and parasitic infections have been carried out (e.g. [8, 9]).

Access to health care is limited, particularly in rural areas. A lack of awareness of entitlements, and informal payment systems, mean that 20–30% of people cannot access primary health care [10].

Another section of the Ministry of Health, the Department of Health and Environment, is responsible for general hygiene and sanitation across all businesses, including food related businesses. The Ministry of Agriculture, Food and Consumer Protection Food Safety Directorate includes the National Food Authority (NFA) which is responsible for official control, risk assessment, and communication. Official control involves the inspection of food production hygiene and certification of hazard analysis critical control point (HACCP) based systems.

Data on the prevalence of hazards in the food supply are limited. Official monitoring programmes for shellfish (algal toxins and *Escherichia coli*) have been in place since 2005 to support exports to the European Union.

### Japan

The major objectives of the Japanese country study were to assess the disease burden from major foodborne diseases in Japan and to analyse the policies on foodborne disease using the FERG framework. The study has now been published [11].

As a pilot study, three major foodborne diseases caused by *Campylobacter* spp., non-typhoid *Salmonella* spp., and enterohaemorrhagic *E*. *coli* (EHEC) were prioritised, based on food poisoning statistics in 2011 and an expert consultation. Firstly the annual incidence was estimated from reported surveillance data adjusted for probabilities of case confirmation and physician visits. The estimated annual incidence was significantly higher than that reported in the routine surveillance data, suggesting a marked underestimation of the magnitude of foodborne diseases.

A series of systematic reviews of disabling sequelae from the three priority diseases was conducted. Subsequently, the estimated incidence was adjusted for food-attributable proportions, which were estimated by an expert elicitation process, similar to that carried out in the Netherlands [12]. Together with the cause-of-death data from vital registration, the disease burden in terms of DALYs was estimated. In 2011, foodborne disease caused by *Campylobacter* spp., non-typhoid *Salmonella* spp., and EHEC led to an estimated 6099, 3145 and 463 DALYs in Japan, respectively. The burden from disabling sequelae was consistently higher than that due to gastroenteritis among the three major foodborne diseases. Data gaps in estimating foodborne disease burden in Japan, in particular population-based data on incidence, were also identified.

Building on the FERG framework, the policy situation analysis provided an overview of the food safety policies, and systems in Japan. As a specific issue to Japan, a rigorous policy situation analysis of the management of risks associated with possible radioactive substances in food, due to the nuclear power plant accident in Fukushima after the Great East Japan Earthquake in 2011, was also completed.

### Thailand

The Thai country study focused on the incidence of diarrheal disease, using data from the National Notifiable Disease Surveillance System maintained by the Bureau of Epidemiology of the Thai Ministry of Public Health. These data were supplemented by information from National Hospital Records (in-patient and out-patient), the National Health and Welfare Survey and community based studies of young children. In this study, the hospital data accessed all three health insurance systems including the universal coverage, social security, and civil servant benefit health insurance. The sharing and interoperability of all three health insurance databases contributed to data reliability and ensured entitlement to the covered health services [13].

Extrapolations from these data sources allowed an estimate of the incidence of acute diarrhea in the community of 10–35 million illnesses in 2009 (for the National Notifiable Disease Surveillance System acute diarrhea is defined as at least 3 loose stools within 24 hours or any abnormal stools (e.g., watery, with mucous, or bloody)). Information on etiology is limited, but the incidence of salmonellosis, cholera, shigellosis, and *E*. *coli* infection were estimated from diagnoses in the National Hospital Record.

In addition, the prevalence of liver fluke infection (*Opisthorchis viverrini*, a locally important foodborne hazard transmitted via fish) and the incidence of rotavirus infection have been estimated.

Food safety regulatory activity in Thailand is led by the Bureau of Food and Water Sanitation, Department of Health, Ministry of Public Health. The popularity of street food has led to the development of a sanitation standard for vendors.

### Uganda

The Ugandan country study established teams to separately address enteric, parasitic and chemical hazards, and source attribution [14]. A detailed situation analysis was prepared, which described the context for food safety in terms of legislation, regulatory authorities, the food supply, production, and consumption. The Ugandan country study was undertaken in conjunction with a project by the Food and Agriculture Organization of the United Nations (FAO) on the use of multi-criteria decision analysis for food safety in Uganda.

Data were collated from surveillance sources (particularly the Health Management Information System administered by the Ministry of Health, and the Central Public Health Laboratory) on acute diarrhea (1.9 million reported outpatient cases in 2012, approximately 5,700 cases per 100,000; case definition: three or more watery stools in 24 hours but not lasting for more than 14 days), cholera, dysentery, brucellosis, hepatitis E and typhoid fever. Parasitic infections are reported as worm infections or intestinal worm infections. Although such infections are very common (approximately 1.8 million outpatient infections reported annually) etiological data are few.

The reliability of these data has improved steadily with increased access to healthcare since 2000. Uganda has undergone a number of reforms that have influenced health service delivery since that date. Among the major reforms, conducted in the early 1990’s was the decentralized governance of districts with attendant devolution of powers to allocate resources and deliver services including health care. Physical access to health facilities for the population living within 5 km of a health facility increased from 49% in 2001 to 72% in 2004 [15].

Other sources of data included Ministry of Agriculture, Animal Industry and Fisheries; Ministry of Trade, Industry and Cooperatives; Ministry of Water and Environment; Ministry of Local Government and Local Authorities and the Research & Academic Institutions.

Of the chemical hazards, the most data were available for aflatoxins, with information on the prevalence of contamination for relevant foods being available. The incidence of hepatocellular carcinoma, an important health outcome of aflatoxin exposure, is also available. Acute poisoning due to methanol in illicit alcoholic beverages is often reported. Despite cassava consumption being high in parts of Uganda, no reports of acute cyanide poisoning, *konzo* or tropical ataxic neuropathy were located.

It was important that both waterborne and foodborne transmission of diseases were included in the Ugandan study, as food safety was not considered to be independent from water safety. It was difficult to generate DALY estimates from the available data—particularly due to the shortage of community level incidence data.

### Findings and Lessons Learned

#### Data gaps

A lack of data prevented DALY calculations in several of the pilot studies. The data gaps included:

- Information to assign etiology for important syndromes such as acute gastrointestinal disease and parasitic infections;
- Data on the incidence of diseases caused by some hazards, particularly chemical hazards; and,
- Limited outbreak and other data on which to base attribution for foodborne transmission.

#### Public and private data sources

In some countries, private hospitals provide a significant proportion of the available healthcare, and may not have the same reporting requirements as public hospitals [16]. Engagement with private hospitals and other facilities to provide a complete picture of the incidence of diseases caused by foodborne hazards may need to be specifically addressed. Data from primary producers and the food industry concerning foodborne hazards can be gathered, but economic implications, particularly for trade, mean that such data should be carefully handled.

#### Foodborne versus waterborne disease

The separation of food and water as exposure vehicles for attribution purposes is often useful as different regulatory agencies may have responsibility for each source. However, at a community level the differentiation between food and water may not be sensible in terms of how risks are managed. These issues should be specifically considered in a national burden study.

#### Situation analysis and knowledge translation

Social scientists, stakeholders and decision makers need to be included in the study team from the earliest stages in order to effectively support knowledge translation and the development of science-based policies. Their involvement includes developing a situation analysis (for an example see [17]), and early and continuous efforts to recognize and incorporate knowledge translation and risk communication to audiences identified in the situation analysis. Differences in experience and perspectives can make collaboration between the social scientists and epidemiological/food safety technical participants challenging.

Knowledge translation and risk communication are usually specialist activities, and require on-going commitment and resources [18]. In order to promote uptake of research results, identified barriers and facilitators are described in the SA/KT/RC Manual.

Barriers to knowledge translation include:

*Limitations resulting from lack of data and information*. Incomplete information, with associated caveats and uncertainty, may prevent clear conclusions for policy being drawn.
*Differing time pressures*. Research may take months or years to complete, whereas policy-makers usually need to produce decisions in much shorter timeframes.
*The weighting of evidence may differ*. Scientists are likely to value data and analysis most highly, whereas policy-makers may be also influenced by personal experience, anecdotal information, political and economic considerations, and other factors.

Knowledge translation can be facilitated by:

*Strong personal relationships between researchers and policy-makers*. Face to face meetings and direct conversations can promote trust and credibility, and support formal written reports.
*Presenting the results of research so that they address risk management questions*. Such questions are best formulated and delivered by policy makers at the commencement of the research, but researchers should always expect to address questions of effectiveness, cost, and high risk groups.

## Discussion

The pilot studies of the national burden of foodborne disease, initiated by WHO, have promoted the importance of such studies amongst the participating countries and disseminated internationally accepted methodology for such estimates. Few DALY estimates were able to be calculated, but this was not unexpected due to data gaps. The first attempt at conducting such studies has identified challenges in both process and information, including the recognition that data collection and analysis, development of situation analysis, and on-going knowledge translation and risk communication require commitment of time and financial resources.

The WHO initiative has provided burden of foodborne disease estimates from global and regional perspectives. These estimates provide context and can fill many of the data gaps for individual countries undertaking foodborne burden of disease studies. In particular, the provision of etiology estimates for syndromic surveillance data, and attribution estimates for foodborne disease will be particularly difficult for studies in developing countries to address individually.

The Global Burden of Disease 2010 Study, undertaken by the Institute for Health Metrics and Evaluation in Seattle USA, covers a broad range of disease and injuries, and has published country specific estimates for these on its website [19]. Foodborne diseases are a subset of these estimates, although estimates are typically not stratified by transmission route. National foodborne disease studies as promoted by WHO and FERG include consideration of the national context in a situation analysis (such as the existing national food control system). In addition, the WHO initiative sought to foster the knowledge translation of burden of disease data into policy through on-going cross-agency communication. Such activities are best undertaken by people from within a country.

National burden of foodborne disease studies, particularly in developing countries, now have an opportunity to fill data gaps, and assign etiology and attribution to the incidence of foodborne diseases, using the data from the WHO initiative to augment local data. Such local data can also be used as a cross check to validate national estimates derived from regional estimates. This should allow the generation of at least preliminary burden estimates to inform national policy. The effective delivery of this information can be guided by the considerations and tools provided in the SA/KT/RC Manual. In the longer term, burden of foodborne disease information should be a fundamental component of a systematic approach to food safety, such as the risk management framework advocated by Codex [20]. Such an approach can enhance both public health and trade.

## Disclaimer

This study was commissioned and paid for by the World Health Organization (WHO). Copyright in the original work on which this article is based belongs to WHO. The authors have been given permission to publish this article.

The findings and conclusions of this report are those of the authors and do not necessarily represent the official views, decisions or policies of the World Health Organization, US Centers for Disease Control and Prevention, the US Government or other institutions listed. FJA is an employee of the US Government. This work was part of his official duties.
